# Is infant arterial stiffness associated with maternal blood pressure in pregnancy? Findings from a UK birth cohort (Baby VIP study)

**DOI:** 10.1371/journal.pone.0200159

**Published:** 2018-07-12

**Authors:** Ka Ying Bonnie Ng, Nigel A. B. Simpson, Janet E. Cade, Darren C. Greenwood, Harry J. Mcardle, Etienne Ciantar, Nisreen A. Alwan

**Affiliations:** 1 Human Development and Health Academic Unit, Faculty of Medicine, University of Southampton, Southampton, United Kingdom; 2 Department of Obstetrics and Gynaecology, Princess Anne Hospital,Southampton, United Kingdom; 3 Department of Women’s and Children’s Health, University of Leeds, Leeds, United Kingdom; 4 Nutritional Epidemiology Group, School of Food Science and Nutrition, University of Leeds, Leeds, United Kingdom; 5 Division of Epidemiology and Biostatistics, School of Medicine, University of Leeds, Leeds, United Kingdom; 6 Rowett Institute of Nutrition and Health, University of Aberdeen, Aberdeen, United Kingdom; 7 Academic Unit of Primary Care and Population Sciences, Faculty of Medicine, University of Southampton, Southampton General Hospital, Southampton, United Kingdom; 8 NIHR Southampton Biomedical Research Centre, University of Southampton and University Hospital Southampton NHS Foundation Trust, Southampton, United Kingdom; Shanghai Institute of Hypertension, CHINA

## Abstract

**Background:**

In adults, arterial stiffness measured by pulse wave velocity (PWV) is regarded as a predictor of cardiovascular disease. Infant vascular development depends on factors related to pregnancy, including maternal blood pressure (BP). This study assessed the association between maternal BP in pregnancy and infant brachio-femoral PWV at age 2–6 weeks.

**Methods:**

The Baby Vascular health and Iron in Pregnancy (Baby VIP) study is a birth cohort which measured PWV and heart rate (HR) in 284 babies in Leeds, UK, at 2–6 weeks after birth. Maternal BP measurements at 12 and 36 weeks gestation was collected from antenatal clinical records. Multivariable linear regression models assessed associations between maternal systolic and diastolic BPs, and BP change from booking to 36 weeks, with infant PWV adjusting for covariables at both mother and baby level.

**Results:**

There was no evidence of an association between infant PWV and maternal systolic BP at booking (adjusted regression coefficient -0.01 m/s per 10mmHg, 95% CI -0.11, 0.14, p = 0.84) or at 36 weeks (adjusted regression coefficient 0.00 m/s per 10mmHg, 95% CI -0.12, 0.11, p = 0.95). Change between 12 and 36 weeks gestation of more than 30 mmHg in systolic BP or 15 mmHg in diastolic BP was also not associated with infant PWV. There was an inverse relationship between infant HR and infant PWV (regression coefficient -0.14 m/s per 10 bpm, 95% CI -0.22, -0.05, p<0.01).

**Conclusions:**

This study has shown no evidence of association between infant PWV at 2–6 weeks of age and maternal BP in early or late pregnancy. Infant HR was inversely associated with infant PWV. Further studies are required to determine the predictors of infant PWV as well as the importance and long term implications of PWV measurements in infants.

## Introduction

Adult aortic pulse wave velocity (aPWV) is an independent predictor for cardiovascular events in adults [1, 2]. The measurement of PWV is a simple, non-invasive and reproducible method to determine arterial stiffness [1], and in adults displaces systolic blood pressure (SBP) or pulse pressure as a predictor for cardiovascular disease mortality. An increase in arterial stiffness can cause premature return of reflected waves in late systole, consequently increasing central pulse pressure and SBP. Increased SBP increases the myocardial oxygen demand by increasing the load on the left ventricle of the heart, and arterial stiffness has been associated with LVH [3, 4]. In both normotensive and hypertensive adults, LVH is another independent predictor of coronary events [5, 6].

The influence of events that occur early in fetal life and their effects on cardiovascular risk in adulthood has been of significant interest [7]. It is well established that reliable measurements of BP are feasible in childhood with normal ranges established [8]. Epidemiological studies have shown a negative association between birth weight and childhood SBP [9–11] as well as an increased risk for the hypertension in adult life for those whose birth weight was low [12]. One study demonstrated a relationship between birth weight and pulse pressure in adolescents, which was measured in the arm over 24 hours as an estimate of arterial stiffness [13]. A subsequent study showed that children with the lowest birth weights have early wave return which depended on the PWV, suggesting an early reduction in the elastic properties of the arteries [13].

Infant and childhood vascular development and BP is in part dependent on factors during pregnancy, including maternal BP [14–17]. The relationship between physiological maternal and infant BP is however largely unknown [18, 19]. One large systematic review has shown a link between maternal chronic hypertension and adverse pregnancy outcomes, including pre-eclampsia, preterm delivery, low birth weight and neonatal death [20]. Children of women with extremes of hypertension in pregnancy, for example, those with pre-eclampsia, have higher BP and an increased risk of vascular events later in life [15, 17]. Determinants of cardiovascular disease, for example BP and PWV may be linked to fetal adaptations later manifest in life; for example, infant PWV has been shown to be associated with birth weight [21, 22]. Maternal BP may be a marker of not only birth weight [23, 24], but also neonatal BP and PWV.

There is strong evidence showing the relationship between extremes of high BP and poor obstetric and neonatal outcomes [20]. However, for women with BP within the ‘normal’ range, the transmission of this risk is less well known and there are fewer studies exploring the maternal BP within this ‘normal’ range and subsequent effects on offspring. Koudsi et al performed a study of 148 babies and found that infant aPWV may be a useful index of infant vascular status, and was better tolerated than BP monitoring; contrary to their hypothesis, they found an inverse relationship between maternal SBP and neonatal aPWV [22].

The aim of this study was to explore the relationship between infant brachio-femoral PWV (bfPWV) at 2–6 weeks of age and maternal systolic and diastolic BP in early and late pregnancy.

## Materials and methods

### Study design

We used data from the Baby VIP (Baby’s Vascular Health and Iron in Pregnancy) birth cohort study. The primary aim of the Baby VIP study was to examine the association between maternal iron status in pregnancy with infant brachio-femoral PWV and birth outcomes [25, 26]. However, given the previous evidence by Koudsi et al that maternal BP is a predictor of infant PWV (22), and the limited published evidence on predictors of infant arterial stiffness, we sought to conduct this analysis. The cohort recruited women aged ≥18 years who gave birth in the Leeds Teaching Hospitals Trust Maternity Unit at a gestational age of ≥34 weeks between February 2012 and January 2013. Participants were recruited from the postnatal wards, and if agreeable to participation, were then asked if a member of the research team could contact them after they were discharged to arrange a home visit within six weeks of discharge. Ethical approval was obtained from the South Yorkshire Committee of the NHS National Research Ethics Service (11/YH/0064) to conduct this study and the University of Southampton Research Ethics Committee (ERGO ID: 20315) to perform this analysis. The study conformed to the 1975 Helsinki Declaration, revised in 1983.

### Outcome measurement

Brachio-femoral (bfPWV) was measured using an oscillometric device (Vicorder, Skidmore Medical). This is a non-invasive approach which has been shown to be relatively independent of operator skills, with reproducible results [27, 28]. The arm cuff was sited at the midpoint of baby’s arm, and the leg cuff at the midpoint of the ipsilateral thigh. Transit time was measured as the time delay between the feet of the proximal and the distal pulse waves. A minimum of two bfPWV readings were taken for each baby, and if there was more than 0.3m/s difference in the readings, a third one was taken. The average PVW reading for each baby was used in the analysis. All measurements for each baby was performed at the same visit, usually separated by a few minutes. The detailed methods for measuring this outcome variable have been described elsewhere [25].

### Exposure measurement

Maternal blood pressures measurements taken by the midwife at each participant’s first antenatal visit (‘booking’) and at 36 weeks gestation were extracted from the antenatal care records. The method of measurement is likely to have followed the National Institute for Health and Care Excellence (NICE) antenatal care guidelines for blood pressure measurement in pregnancy [29]. A cut off of ≥140 mm Hg and ≥ 90 mm Hg were used to indicate ‘hypertension’ for systolic and diastolic measurements respectively. These cut-off values were based on the Hypertension in Pregnancy Guideline from NICE [30]; they class a BP of 140/90 to 149/99 mm Hg as mild hypertension, BP of 150/100 to 159/109 mm Hg as moderate hypertension, and BP of 160/110 mm Hg or higher as severe hypertension. However, hypertension in pregnancy can also be diagnosed as a relative rise above measurements obtained at booking; the definition for this is either a rise in systolic BP of >30 mmHg or rise in diastolic BP of >15 mm Hg above the BP at booking [31]. Therefore, we also calculated the change in systolic and diastolic blood pressure measurements between booking and at 36 weeks gestation.

### Covariable assessment

The Index of Multiple Deprivation (IMD) is derived from GeoConvert which uses UK census data (geoconvert.mimas.ac.uk). Covariables including birth weight, gestational age, parity, maternal height, weight ethnicity, smoking status, pregnancy complications (pre-eclampsia, gestational diabetes), blood pressure measurements and oral iron supplement intake were extracted from the clinical health records. Infant heart rate was recorded by the Vicorder device and used in the analysis if there were at least two measurements documented in the database. The average of these two independent readings was used in the statistical models.

### Statistical methods

Statistical analysis was performed using Stata version 14 (2016; College Station, Tex., USA). We assessed the relationship between infant PWV as the outcome variable and maternal BPs as the predictors using regression analyses. Initially univariable (no adjustment for covariables) analyses were undertaken, followed by multiple linear regression models that first adjusted for the possible confounders, then adjusted for the aformentioned confounders and infant heart rate. The confounders adjusted for included baby covariables (gestational age (days), birthweight (grams), feeding status, age at measurement of PWV, baby’s position, sleep/wake phase and sex) and maternal covariables (maternal age, smoking status, ethnicity, BMI, IMD score, parity and presence of gestational diabetes). A statistical significance level of 5%, with 95% confidence intervals was used in the regression models. In addition, we planned to use multiple linear regression models to assess infant heart rate (HR) as a potential mediator of any potential significant association between infant PWV and maternal BP. The infant HR was taken from a mean of two readings.

### Study power

The original sample size calculation for this cohort is described elsewhere (25). Retrospective study power calculation for this analysis was performed. For a study power of 80% at a statistical significance level of 0.05, using the mean (6.7 m/s) and standard deviation (1.3 m/s) of PWV from this study, a sample size of 270 mother-baby pairs is required to detect a difference of 0.44 m/s in bfPWV.

## Results

A total of 362 mother-baby pairs were recruited into the study, and 284 (79%) babies had a PWV measurement at a follow up home visit. A total of 276 mother-baby pairs had infant bf-PWV measurements and maternal BP readings at booking and at 36 weeks gestation. Mean infant PWV was 6.7 m/s (95% CI 6.5–6.8). At booking the mean maternal systolic and diastolic BP readings were 112 mmHg (95% CI 110–113) and 67 mmHg (95% CI 66–68). A 36 weeks gestation, the mean maternal systolic and diastolic BP readings were 116 mmHg (95% CI 115–118) and 71 mmHg (95% CI 70–72). Table 1 describes the characteristics of the participants, categorised by group with maternal systolic BP reading of ≥ 140 mmHg or <140mmHg.

**Table 1:** Baby VIP Study sample characteristics categorised by maternal systolic blood pressure at 36 weeks gestation.

Characteristic	Maternal systolic BP ≥ 140 mmHg at 36 weeks (n = 14)		Maternal systolic BP <140 mmHg at 36 weeks (n = 262)		P value
***Mean*, *Standard Deviation***					
**Maternal age, yrs** (n = 276)	30.5	6.4	31.0	5.5	0.7
**Maternal BMI, kg/m**^**2**^ (n = 273)	32.6	8.0	26.2	5.8	<0.001
**Age of PWV measurement, days** (n = 275)	27.1	0.5	24.4	0.4	0.1
**Baby’s birthweight, g** (n = 276)	3069	559	3381	609	0.06
**Baby’s heart rate, beats/min** (n = 207)	117	23	117	19	1.0
**Baby’s PWV, mmHg** (n = 276)	6.4	1.1	6.7	1.3	0.4
**Baby’s gestational age at birth, days (n = 276)**	274	14	278	13	0.2
***N (%) of cases*, *95% CI***					
**Parity** (n = 276)					
Nulliparous (n = 143)	8 (57.1)	28.9, 82.3	135 (51.5)	45.3, 57.7	1.0
Multiparous (n = 133)	6 (42.9)	17.7, 71.1	127 (48.5)	42.3, 54.7	
**Smoking status**					
Never smoked (n = 153)	5 (35.7)	12.8, 64.9	148 (56.5)	50.2, 62.6	0.3
Smoker (n = 36)	2 (14.3)	1.8, 42.8	34 (13.0)	9.2, 17.7	
Ex-smoker (n = 87)	7 (50.0)	23.0, 77.0	80 (30.5)	25.0, 36.5	
**Ethnicity**					
White British (n = 216)	12 (85.7)	57.2, 98.2	205 (77.9)	72.3, 82.7	0.9
Other ethnicity (n = 60)	2 (14.3)	1.8, 42.8	58 (22.1)	17.3, 27.7	
**Presence of diabetes**					
Yes (n = 4)	0 (0)	0, 23.2	4 (1.5)	0.4, 3.9	0.6
No (n = 272)	14 (100)	76.8, 1	258 (98.5)	96.1, 99.6	
**Method of feeding**					
Breastfeeding (n = 120)	5 (35.7)	12.8, 64.9	115 (43.9)	37.9, 50.2	0.7
Bottle feeding (n = 106)	7 (50.0)	23.0, 77.0	99 (37.8)	32.0, 44.0	
Mixed feeding (n = 50)	2 (14.3)	1.8, 42.8	48 (18.3)	13.8, 23.5	

Complete cases for exposures, outcomes, confounders and mediators. Where there was a continuous variable, a t-test was used for analysis, and where there was a categorical variable, a chi-squared test was used.

The results for the univariable analyses and multiple linear regression analyses, adjusting for confounders and mediators are shown in Tables 2–4. In unadjusted analyses, there was no evidence of significant associations between infant bfPWV and maternal systolic or diastolic BP at either booking or 36 weeks gestation (Table 2). For every 10mmg increase in systolic BP at booking, there was no evidence of a statistically significant change in the bfPWV (adjusted regression coefficient 0.01, 95% CI -0.11, 0.14, p = 0.84). For every 10mmg increase in diastolic BP at booking, there was no evidence of a statistically significant change in the bfPWV (adjusted regression coefficient 0.03, 95% CI -0.14, 0.20, p = 0.72). For every 10mmg increase in systolic BP at 36 weeks gestation, there was no evidence of a statistically significant change in the bfPWV (adjusted regression coefficient 0.00, 95% CI -0.12, 0.11, p = 0.95). For every 10mmg increase in diastolic BP at 36 weeks gestation, there was no evidence of a statistically significant change in the bfPWV (adjusted regression coefficient 0.02, 95% CI -0.12, 0.15 p = 0.80).

**Table 2:** Associations between infant pulse wave velocity and maternal blood pressure as a continuous variable in the Baby VIP Study.

Predictor	Difference in infant bfPWV (m/s), unadjusted			Difference in infant bfPWV (m/s), adjusted for confounders			Difference in infant bfPWV (m/s), adjusted for confounders and infant HR		
	Mean difference	95% CI	P-value	Mean difference	Adjusted 95% CI	P-value	Mean difference	Adjusted 95% CI	P-value
Maternal systolic BP at booking (mmHg)*	0.03	-0.09, 0.15	0.61	0.01	-0.11, 0.14	0.84	0.03	-0.12, 0.18	0.72
Maternal diastolic BP at booking (mmHg)*	0.07	-0.10, 0.23	0.43	0.03	-0.14, 0.20	0.72	0.00	-0.16, 0.22	0.77
Maternal systolic BP at 36 weeks (mmHg)*	0.03	-0.08, 0.13	0.59	0.00	-0.12, 0.11	0.95	0.04	-0.08, 0.17	0.49
Maternal diastolic BP at 36 weeks (mmHg)*	0.02	-0.11, 0.15	0.75	0.02	-0.12, 0.15	0.80	0.03	-0.11, 0.18	0.66

*Mean difference in PWV reflects 10mmHg change in BP

Multiple linear regression models were used to determine associations. Confounders include maternal factors (maternal age, smoking status, ethnicity, BMI, IMD score, parity presence of gestational diabetes) and baby factors (gestational age, birth weight, feeding status, age at measurement of PWV, baby’s position, sleep/wake phase and sex). Infant HR was assessed as a potential mediator.

**Table 3:** Associations between infant pulse wave velocity and maternal hypertension (systolic BP ≥ 140 and diastolic BP ≥ 90) in the Baby VIP Study.

Predictor	Difference in infant bfPWV (m/s), unadjusted			Difference in infant bfPWV (m/s), adjusted for confounders			Difference in infant bfPWV (m/s), adjusted for confounders and infant HR		
	Mean difference	95% CI	P-value	Mean difference	Adjusted 95% CI	P-value	Mean difference	Adjusted 95% CI	P-value
**Maternal systolic BP ≥ 140 mmHg at booking (n = 7)**	-0.21	-1.15, 0.73	0.65	-0.19	-1.14, 0.75	0.69	-0.16	-1.11, 0.80	0.74
**Maternal diastolic BP ≥ 90 mmHg at booking (n = 6)**	0.94	-0.13, 2.02	0.09	0.86	-0.21, 1.93	0.12	1.09	-0.19, 2.20	0.05
**Maternal systolic BP ≥ 140 mmHg at 36 weeks (n = 14)**	-0.30	-1.01, 0.42	0.41	-0.37	-1.11, 0.37	0.33	0.20	-0.66, 1.05	0.65
**Maternal diastolic BP ≥ 90 mmHg at 36 weeks (n = 8)**	-0.09	-1.02, 0.85	0.85	-0.10	-1.04, 0.83	0.83	0.14	-0.88, 1.17	0.78

Multiple linear regression models were used to determine associations. Confounders include maternal factors (maternal age, smoking status, ethnicity, BMI, IMD score, parity and presence of gestational diabetes) and baby factors (gestational age, birth weight, feeding status, age at measurement of PWV, baby’s position, sleep/wake phase and sex). Infant HR was assessed as a potential mediator.

**Table 4:** Associations between infant pulse wave velocity and a significant change in maternal BP from booking to 36 weeks gestation (change in systolic BP >30mmHg and change in diastolic BP >15mmHg) in the Baby VIP Study. Out of 276 subjects, 13 (5%) had a change in systolic BP >30 mmHg and 35 (13%) had a change in diastolic BP >15mmHg.

Predictor	Difference in infant bfPWV (m/s), unadjusted			Difference in infant bfPWV (m/s), adjusted for confounders			Difference in infant bfPWV (m/s), adjusted for confounders and infant HR		
	Mean difference	95% CI	P-value	Mean difference	Adjusted 95% CI	P-value	Mean difference	Adjusted 95% CI	P-value
Change in systolic BP >30mmHg** **(n = 13)**	-0.34	-1.08, 0.40	0.37	-0.52	-1.26, 0.22	0.16	-0.16	-1.18, 0.86	0.76
Change in diastolic BP >15mmHg** **(n = 35)**	0.21	-0.26, 0.68	0.37	0.19	-0.28, 0.66	0.42	0.25	-0.28, 0.78	0.36

** Change in blood pressure from BP measurement at booking to measurement at 36 weeks gestation.

Confounders include maternal factors (maternal age, smoking status, ethnicity, BMI, IMD score, parity and presence of gestational diabetes) and baby factors (gestational age, birth weight, feeding status, age at measurement of PWV, baby’s position, sleep/wake phase and sex). Infant HR was assessed as a potential mediator.

There was no evidence of an association between infant bfPWV and maternal systolic hypertension (≥140 mmHg) at booking (adjusted regression coefficient -0.19, 95% CI -1.14, 0.75, p = 0.69) or at 36 weeks gestation (adjusted regression coefficient -0.37, 95% CI -1.11, 0.37, p = 0.33) (Table 3). There was no evidence of an association between infant bfPWV and maternal diastolic hypertension (≥90 mmHg) at booking (adjusted regression coefficient 0.86, 95% CI -0.21, 1.93, p = 0.12) or at 36 weeks gestation (adjusted regression coefficient –0.10, 95% CI -1.04, 0.83 p = 0.83) (Table 3). There was also no evidence of an association between infant bfPWV and a change in systolic BP from booking to 36 weeks (>30 mmHg increase) (adjusted regression coefficient -0.52, 95% CI -1.26, 0.22, p = 0.16) or a change in diastolic BP from booking to 36 weeks (>15 mmHg increase) (adjusted regression coefficient 0.19, 95% CI -0.28, 0.66, p = 0.42) (Table 4).

There was no evidence of significant associations between maternal BP parameters and bfPWV, when adjusting for infant HR as well as confounders (Tables 2–4).

There was an inverse relationship between infant HR and infant PWV. The regression coefficient of the relationship is a PWV of -0.14m/s per every 10bpm increase in infant HR, 95% CI -0.22, -0.05, p<0.01 (Adjusted regression coefficient -0.11, 95% CI -0.20, -0.02, p = 0.01). The adjusted model included maternal age, smoking status, ethnicity, BMI at booking, parity, IMD score, presence of gestational diabetes, baby’s gestational age, birthweight, gender, type of feeding, age at PWV measurement, position and whether asleep or awake at the time of measurement.

## Discussion

This study has shown that there is no evidence of any associations between infant PWV and maternal hypertension in pregnancy, maternal BP as a continuous variable in early and late pregnancy, or change in maternal BP (>30 mmHg in SBP or 15 mmHg in DBP between early and late pregnancy). Infant HR was inversely associated with infant PWV.

These findings do not support Koudsi et al’s study findings which showed an inverse relationship between maternal SBP at 28 weeks gestation and neonatal aortic PWV [22]. There are some potential reasons for the difference in our findings to this previous study. Koudsi et al’s study was performed on 148 mothers and neonates, it looked at the PWV in neonates in the first few days of life only, and they only investigated the association with maternal SBP at 28 weeks gestation [22]. They performed a reproducibility study on 30 infants only, and concluded that the aortic PWV method was reliable, giving similar readings at baseline and up to 3 days later. They used this to justify that a mean PWV result was not necessary. Our study expands on the study done previously by Koudsi et al [22]; it includes a larger sample size of mothers and infants, we take the mean PWV measurements from two or three readings (where the mean measurement is used for analysis), the PWV is measured between 2–6 weeks of age and we include associations with maternal BP measurements at booking, and at 36 weeks gestation. We also assessed the association of an increase in the systolic and diastolic BP from booking to 36 weeks gestation with infant PWV. We performed a sensitivity analysis where we adjusted for the infant HR in association with maternal BP and infant PWV. Infant HR cannot be considered a true confounder as it cannot directly influence maternal BP, and therefore was not included in the main model.

Our study reports an average bf-PWV of 6.7 m/s, which is higher than that reported by other studies which have assessed the aortic PWV in the immediate postnatal period [22] and those in slightly older infants and young children [32]. This may be explained by the use of peripheral arteries (namely the brachial and the femoral) in our study, which are ‘muscular arteries’, containing fewer elastic fibers and more smooth muscle cells than conducting ‘elastic arteries’ such as the aorta. Less elastic fibers within the tunica media of an artery will mean less distensability when a pulse wave is passed, and an increased arterial stiffness, reflected by the higher PWV in this study. In adults, the brachio-ankle PWV is higher than the carotid-femoral PWV by approximately 20% [33].

Environmental exposure in utero is known to have significant impacts on the developmental health and wellbeing of the offspring later on in life [7, 34]. In the human, the hemochorial placenta allows the fetal tissues to interact directly with the mother’s blood, allowing rapid diffusion of nutrients and oxygen to the fetus; thus, effects on maternal vasculature and placental vascular function have the potential to be translated to the fetus. There is also evidence to show that infant and childhood vascular development is dependent on factors related to pregnancy including BP [14–17]. Despite convincing evidence to suggest a plausible relationship, we have shown no associations between maternal hypertension, or maternal blood pressure antenatally and infant PWV. This may be a ‘true no association’ or may be type 2 error due to insufficient statistical power. There may be non-differential information bias, for example, most babies could not remain completely still during the PWV measurement causing random measurement error. Additionally, our study has used a ‘healthy’ population, mostly with normal blood pressures and so the effect of ‘true pathology’ (for example higher degrees of hypertension), on infant PWV may remain concealed in this study.

It remains unclear how maternal blood flow and the integrity of the vasculature affects arterial growth and development in the neonate. Mechanical properties of the arterial walls depend on extracellular matrix components produced by smooth muscle cells [35]. Elastin and proteins including lysyl oxidase, fibrillians and fibulins, have the highest expression during the late embryonic and neonatal period (with minimal expression during adulthood) [35] and allow arteries to maintain sufficient blood pressures.

In this study, we showed an inverse relationship between infant heart rate and PWV, which is contrary to positive relationship between the two variables in the analysis by Koudsi et al [22]. In adults, it is recognised that arterial stiffness (higher PWV) is associated with a higher resting heart rate [36], however, this relationship in infants is not widely explored and different physiological mechanisms in the newborn may explain our findings. There are complex neurohumoral and metabolic responses maintaining blood pressure and heart rate in the newborn. For example, in later gestations in the fetus and the newborn, there is decreasing HR as blood pressure rises due to baroreceptor activity, which is particularly sensitive to changes in BP in the aortic arch and carotid sinuses.

There are some limitations of our study. We used bf-PWV measurements, rather than aortic PWV or other centrally located PWV measurements (e.g. carotid artery), which may be a potential source of error [37]. The Vicorder device has been used in this study to assess arterial stiffness using bfPWV. In a study to compare PWV measurements using the Vicorder and the SphygmoCor devices in 156 children, Keehn et al illustrate that the Vicorder gives good repeatability in children with close correlation of values obtained using the brachio-femoral and carotid-femoral paths, however, the latter values were only moderately correlated with the those obtained using the ShygmoCor system making the Vicorder a potentially less accurate method of assessing PWV in children [38]. However, given its ease of use and reproducibility, differences are still likely to discriminate between groups with differing arterial stiffness, particularly if utilised in population studies using relatively large study numbers.

We used the measurement of distance between the two arterial sites as a proxy for the distance that the pulse wave travels, which may be a source of error. This potential source of error can be overcome by using magnetic resonance imaging (MRI) to measure PWV; MRI allows accurate measurement of the path length of the pulse wave within the aorta, even in the presence of a tortuous vessel [39]. We recognise infant BP as a recognised determinant of infant PWV [22], however, we did not measure infant BP in this study so did not adjust for this variable in our model. We also used routinely-recorded maternal BP measurements performed as part of antenatal care at specified time points, and acknowledge that this method of assessment may be less rigorous than if the measurements were taken as part of a research study protocol.

At present, the predictors of infant PWV remain largely unknown. There have been several observational studies which have tried to address potential influencers of infant PWV. Infant PWV has been shown to be affected by birth weight [21, 22]; small for gestational age and lower birthweights has been linked to a lower infant PWV (lower degree of arterial stiffness) [21]. Maternal anaemia prior to 20 weeks gestation has been associated with an increased infant PWV [25]. Neonates of mothers with higher haemoglobin A1c have a higher PWV [40].

## Conclusions

Our study does not show any association between maternal hypertension or antenatal blood pressure measurements and infant PWV. However, this area warrants further investigation, especially as these findings differ for a previous study. Longer term follow up of these babies through to childhood and adult life may help us assess the usefulness of infant PWV as a non-invasive tool in predicting cardiovascular events later in life. This study has demonstrated feasibility of taking bf-PWV measurements in a relatively large sample of infants in our population.
